# Gene Expression Profiling Reveals New Potential Players of Gonad Differentiation in the Chicken Embryo

**DOI:** 10.1371/journal.pone.0023959

**Published:** 2011-09-09

**Authors:** Gwenn-Aël Carré, Isabelle Couty, Christelle Hennequet-Antier, Marina S. Govoroun

**Affiliations:** 1 Physiologie de la Reproduction et des Comportements UMR 6175, INRA- CNRS-Université F. Rabelais de Tours-Haras Nationaux, Nouzilly, France; 2 Unité de Recherche Avicole, INRA, Nouzilly, France; Centro Cardiologico Monzino, Italy

## Abstract

**Background:**

In birds as in mammals, a genetic switch determines whether the undifferentiated gonad develops into an ovary or a testis. However, understanding of the molecular pathway(s) involved in gonad differentiation is still incomplete.

**Methodology/Principal Findings:**

With the aim of improving characterization of the molecular pathway(s) involved in gonad differentiation in the chicken embryo, we developed a large scale real time reverse transcription polymerase chain reaction approach on 110 selected genes for evaluation of their expression profiles during chicken gonad differentiation between days 5.5 and 19 of incubation. Hierarchical clustering analysis of the resulting datasets discriminated gene clusters expressed preferentially in the ovary or the testis, and/or at early or later periods of embryonic gonad development. Fitting a linear model and testing the comparisons of interest allowed the identification of new potential actors of gonad differentiation, such as Z-linked *ADAMTS12*, *LOC427192* (corresponding to NIM1 protein) and *CFC1*, that are upregulated in the developing testis, and *BMP3* and Z-linked *ADAMTSL1*, that are preferentially expressed in the developing ovary. Interestingly, the expression patterns of several members of the transforming growth factor β family were sexually dimorphic, with inhibin subunits upregulated in the testis, and bone morphogenetic protein subfamily members including *BMP2*, *BMP3*, *BMP4* and *BMP7*, upregulated in the ovary. This study also highlighted several genes displaying asymmetric expression profiles such as *GREM1* and *BMP3* that are potentially involved in different aspects of gonad left-right asymmetry.

**Conclusion/Significance:**

This study supports the overall conservation of vertebrate sex differentiation pathways but also reveals some particular feature of gene expression patterns during gonad development in the chicken. In particular, our study revealed new candidate genes which may be potential actors of chicken gonad differentiation and provides evidence of the preferential expression of BMPs in the developing ovary and Inhibin/Activin subunits in the developing testis.

## Introduction

Sex determination in birds is controlled by a genetic ZZ/ZW system in which the female is heterogametic. The avian sex determining mechanism has not been completely elucidated to date, and no homolog of the mammalian sex determining gene, SRY, has been found in birds. However, a recent study suggested that the *DMRT1* gene (Doublesex and Mab-3 Related Transccription factor 1), belonging to the zinc finger-like DNA-binding motif (DM) transcription factor family and located on the Z chromosome, acts as a male sex determining factor [1]. Although *DMRT1* is essential for testis differentiation, another Z-linked gene may operate upstream of *DMRT1* in the avian male determining pathway. It is also possible that a still unidentified female W-linked determinant may be required for ovary differentiation. Nevertheless, overexpression of the W-linked candidate sex determinant *HINTW* (also known as *ASW* or *WPKCI*) in male embryos does not induce ovary differentiation [2]. Gonad development in birds is in many aspects similar to that of mammals. As in mammals, the avian primordial gonads develop from the intermediate mesoderm, on the ventromedial surface of the embryonic kidney (mesonephros). Germ cells colonize the genital ridge by day 3.5 and the formation of primitive sex cords occurs by day 5 [3]. The gonads of ZZ and ZW embryos remain indistinguishable until day 6.5–7 (stage 30 of the Hamburger and Hamilton classification [4]), when the first histological signs of sex differentiation become visible. This process includes the thinning of the cortex and development of testicular cords enclosing germ cells in ZZ gonads. In the ZW left gonad, somatic and germ cells proliferate in the cortex, which thickens considerably, while the cords of the medulla become vacuolated, forming lacunae. The germ cells of the left ZW gonad enter in meiosis asynchronously from day 15 [5], [6], [7]. The meiotic entry of female germ cells is then completed before hatching. Comparative studies have demonstrated that genes involved in the sex differentiation pathway are conserved in vertebrates; nevertheless their roles, their regulation and their expression time-windows during this process can differ. For instance, similar to mammals, *AMH* and *SOX9* are upregulated in the chicken testis during gonad development. However, in contrast to mammals, *AMH* is also expressed in embryonic female gonads in chicken but at a lower level, and the onset of its expression precedes that of *SOX9* [8]. Since both proteins are found in Sertoli cells, *SOX9* may be involved in maintaining AMH expression [9]. In addition, the chicken model presents two particular features. The first, in contrast to mammals, gonad differentiation is sensitive to exogenous hormonal manipulation. Indeed, inhibition of *CYP19A1* causes female to male sex reversal [10], while estrogen treatment of ZZ embryos leads to feminization of left gonads and regression of right gonads [11], [12]. These experiments revealed a crucial role of estrogen production during avian ovary development and the fundamental role of regulation of *CYP19A1* expression. There is a lack of detailed knowledge concerning molecular mechanisms leading to the activation of *SOX9* and *AMH* expression in male gonads and to *FOXL2* and *CYP19A1* expression in female gonads in birds. Furthermore, the target and downstream pathways of these genes are poorly understood. Another particular feature of chicken gonadal differentiation is its left-right (L-R) asymmetry in the female. Gonadal development in male birds results in the formation of two functional testes. In female birds, only the left gonad becomes a functional ovary whereas the right reproductive system regresses. Recent studies revealed that left sided expression of *PITX2*, *RALDH2* and *SF1* leads to only the left sided expression of estrogen receptor alpha (*ESR1*) [11], [13], [14]. Estrogen production by embryonic ZW gonads thus promotes left cortical development via ESR1 and regression of the right gonad, where the level of expression of *ESR1* is dramatically decreased. In ZZ gonads which do not produce estrogens, the asymmetry remains silent. However, the other actors of complex molecular mechanisms of gonad asymmetry and the factors involved in gonad regression remain to be established. In the light of this current knowledge our study aimed to identify new candidate genes involved in male and female gonad differentiation and in L-R gonad asymmetry in birds. We therefore developed a large-scale real-time reverse transcription polymerase chain reaction (RT-PCR) approach on 110 selected candidate genes for investigation of their expression profiles during gonad differentiation and development in the chicken embryo. Selection of the set of candidate genes was mainly based on the literature. It included transcription factors, receptors, signalling molecules and enzymes known to be involved in gonad development in other vertebrate species, and more generally in reproduction and embryogenesis. A linear model was fitted and contrasts of interest were tested. The expression profiles were also explored using a hierarchical clustering method. Our study revealed many genes that behave differently between sexes and/or stage-specific groups including potential new actors of sex gonad differentiation and establishment of L-R gonad asymmetry.

## Results

### Overal analysis

The raw data set is presented in an online supplement (Table S1). For each date of sampling, the relative expression of each gene was obtained in two independent pools of gonads (biological replicates). To determine the consistency of our finding, we calculated correlation coefficients (r) of biological replicates. The correlation values of biological replicates ranged from 0.87 to 0.99 (Figure S1).

**Figure 1:**
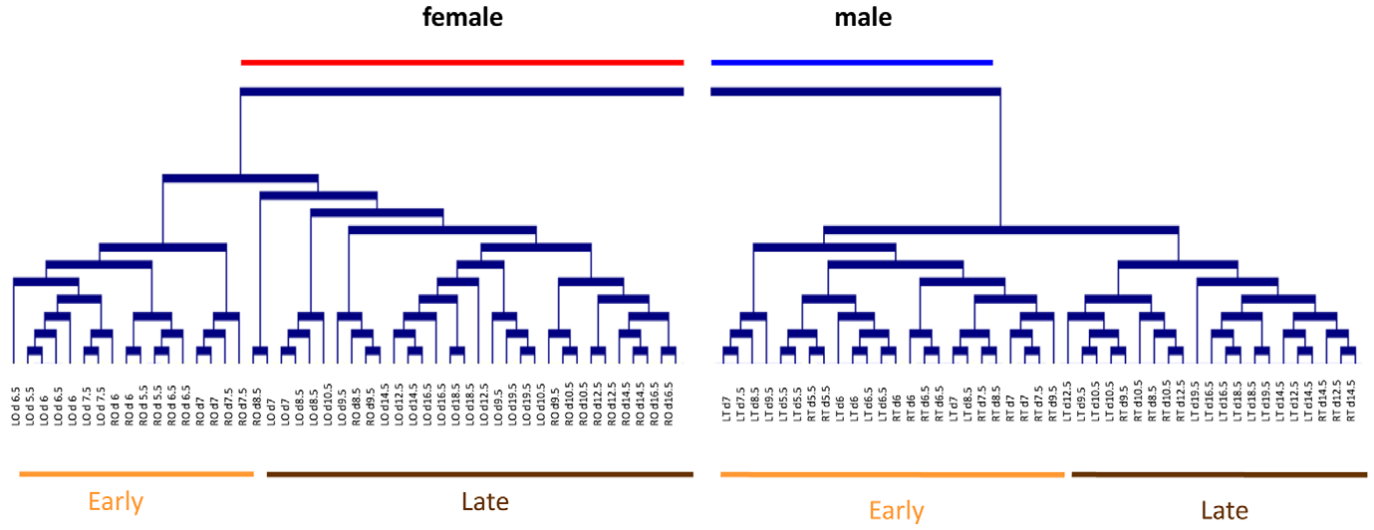
Hierarchical Clustering of the biological samples. Hierarchical clustering was performed using complete linkage algorithm applied on the similarity matrix based on the Pearson correlation coefficient. LO = left ovary; RO = right ovary; LT = left testis; RT = right testis.

Based on the efficiency and specificity of Real-time RT-PCR, 110 genes were finally selected to identify differentially expressed genes and to cluster genes into groups with similar expression profiles. To assess the biological relevance of the genes assayed, we first performed hierarchical clustering of the samples (Figure 1) that generated two large groups corresponding to male and female gonads. In each group, cluster analysis discriminated two subgroups of samples, one that contained the early samples (day 5.5 to day 7.5 for female gonads and day 5.5 to day 8.5 for male gonads), and another that contained the later samples (day 8.5 to day 19.5 for female gonads and day 9.5 to day 19.5 for male gonads). Based on these findings, for the statistical analysis we chose to discriminate two periods during development: before day 7.5 and from day 8.5 referred to as the earlier and later periods respectively. Within these groups, almost all the biological duplicates were clustered together, showing the efficiency of the method and confirming the relevance of the samples and genes assayed. Furthermore, within these classes the samples were grouped according to whether they came from a right or left gonad, suggesting that there is differential expression of several genes between the two sides.

### Differential analysis and gene cluster analysis

**Figure 2:**
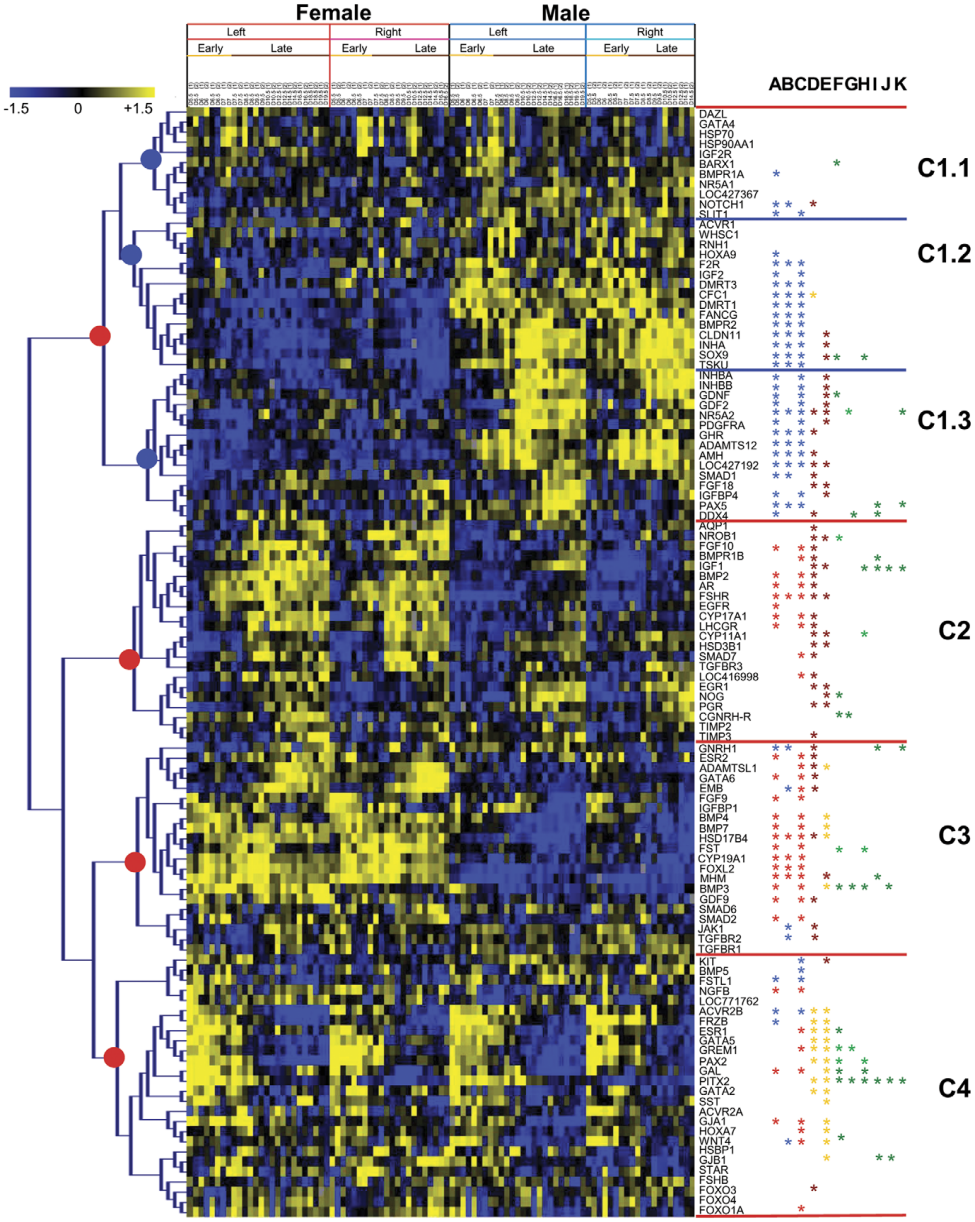
Hierarchical clustering of 110 gene expression patterns in right and left male and female chicken gonads. Each row represents a gene, and each column represents a sample. The samples at the top of the heatmap are set out according to sex (male and female), according to side (left or right) and according to development stage. Each cell in the matrix corresponds to an expression level, with blue for underexpression, yellow for overexpression, black for gene expression close to the median (see color scale) and grey for missing values. Genes are grouped according to their expression pattern across all samples using hierarchical clustering based on the Pearson correlation coefficient as a similarity matrix between genes, and complete linkage algorithm. Particular clusters are quoted on the right side of the picture. Differential analysis was performed with the Limma R package. Genes are considered differentially expressed if the adjusted p values by the Benjamini and Hochberg's method are below a threshold fixed at 0.05 for the contrast tested: A) Analysis of all left female samples vs all left male samples. B) Analysis of early left female samples vs early left male samples. C) Analysis of late left female samples vs late left male samples. D) Analysis of early left female samples vs late left female samples. E) Analysis of early left male samples vs late left male samples. F) Analysis of left female samples vs right female samples. G) Analysis of left vs right early female samples. H) Analysis of left vs right later female samples. I) Analysis of left male samples vs right male samples. J) Analysis of left vs right early testis samples. K) Analysis of left vs right later testis samples. Red asterisks indicate overexpression in female gonads, blue asterisks indicate overexpression in male samples, brown asterisks indicate overexpression in late group of samples, orange asterisks indicate overexpression in early group of samples, dark green asterisks indicate overexpression in left gonads and light green asterisks indicate overexpression in right group of samples.

Differential analysis was performed using a linear model. Lists of differentially expressed genes for each comparison were obtained after adjustment for multiple testing [15]. This analysis showed that 80.9% of genes displayed a statistically significant difference in their expression profiles in at least one of the comparisons. In order to group genes according to the similarity of their expression patterns, we also performed hierarchical clustering of genes with samples ordered on the basis of the developmental stage (from day 5.5 to day 19.5) (Figure 2).

As shown in Figure 2, analysis of the dendrogram revealed that genes were clustered in four main groups. The first group corresponded to genes with the highest relative expression (RE) in the testis (node 1). The second group corresponded to genes with a highest RE in later samples associated with preferential RE in the ovary (node 2). The third group contained genes with the highest RE in the ovary (node 3) while the last group corresponded to genes with the highest RE in early samples (node 4). Clusters were strongly supported by the existence of significant differences in gene expression between samples in particular comparisons, indicated by colored asterisks on the right of Figure 2.

**Figure 3:**
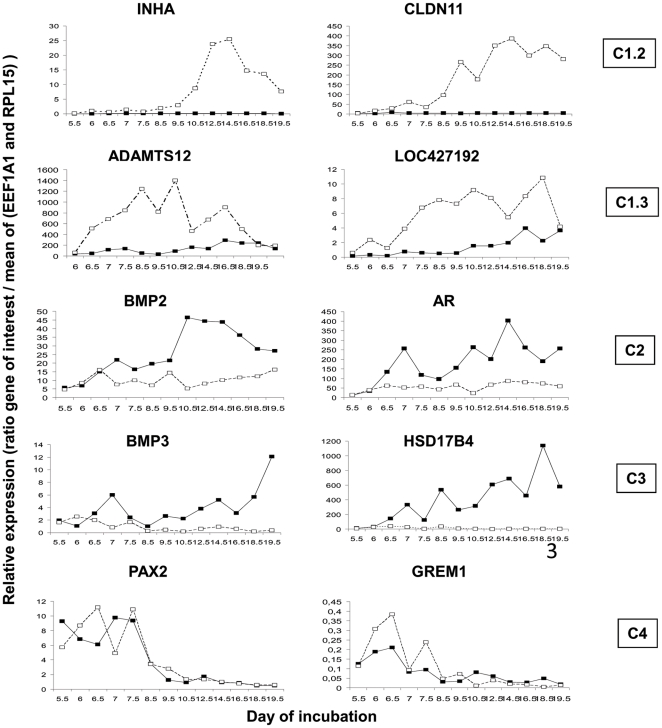
Expression profiles of some representative genes from the four main clusters (C1 to C4) identified during male and female sex differentiation. Cluster C1.2: *INHA* (inhibin alpha) and *CLDN11* (claudin 11 (oligodendrocyte transmembrane protein)); cluster C1.3: *ADAMTS12* (ADAM metallopeptidase with thrombospondin type 1 motif, 12) and *LOC427192* (similar to hypothetical protein MGC42105); cluster C2: *BMP2* (bone morphogenetic protein 2) and *AR* (Androgen receptor); cluster C3: *BMP3* (bone morphogenetic protein 3) and *HSD17B4* (Hydroxysteroid (17-beta) dehydrogenase 4); Cluster C4: *PAX2* (paired box 2) and *GREM1* (gremlin 1). For each histogram, female samples are represented by black squares and black lines and male samples by empty squares and dotted line. Results are represented with an arbitrary scale as the ratio between the expression of the specific gene and the mean expression of *EEF1A1*and *RPL15*. Each square represents the mean of two different measurements from different biological samples for the same male or female population and the same sampling date.

Cluster C1 (Figure 2) contained forty-one genes, the majority of them displaying a preferential RE in the testis compared to the ovary. This cluster could be divided into three subgroups: C1.1, C1.2 and C1.3. The genes from subgroup C1.1 were not characterized by any similarity in their expression profiles. However, several genes showed sexual dimorphism (*BMPR1A*, *NOTCH1* and *SLIT1*), among which *SLIT1* was upregulated in the testis compared to the ovary in samples from day 8.5 (“later” period) while the RE of *NOTCH1* was higher in the testis samples from the earlier period. In the second subgroup, (C1.2), ten genes were significantly upregulated in the testis compared to the ovary in both time periods (*F2R*, *DMRT3*, *CFC1*, *DMRT1*, *FANCG*, *BMPR2*, *CLDN11*, *INHA*, *SOX9* and *TSKU*) whereas *IGF2* was upregulated in the testis only in the later period. In addition, the RE of three of these genes increased during testis development (*CLDN11*, *INHA* and *SOX9*). Interestingly, among genes with preferential higher RE in the testis, one gene, (*CFC1*) was also downregulated during ovarian development. Representative expression profiles of this subcluster are shown in Figure 3 (See *INHA* and *CLDN11*). The third subcluster (C1.3) includes genes whose RE was also globally higher in the testis, and in addition, was increased during gonad development. Representative expression profiles of this subcluster are shown in Figure 3 (See *ADAMTS12* and *LOC427192*). Similar to the *AMH* pattern, five genes demonstrated preferential testicular expression in both time periods (*NR5A2*, *GHR*, *ADAMTS12*, *LOC427192* (corresponding to NIM1) and *PAX5*). Six genes were significantly more highly expressed in the testis samples than in the ovary samples in the later period (*INHBA*, *INHBB*, *GDNF*, *GDF2*, *PDFGRA*, and *IGFBP4*) and one gene (*SMAD1*) was preferentially expressed in the testis samples only in the early period. The RE of three genes (*NR5A2*, *LOC427192* (corresponding to NIM1) and *FGF18*) was higher in the later period compared to the earlier period in both sexes. Furthermore, the RE of six genes increased only during testis development (*INHBA*, *INHBB*, *GDNF*, *GDF2*, *PDGFRA* and *IGFBP4*), while the RE of *GHR*, *AMH*, *SMAD1* and *DDX4* increased only during ovary development.

Cluster C2 (Figure 2) was characterized by genes whose RE was globally increased during ovary development. Indeed, seventeen genes were upregulated during ovary development between the earlier and later periods (*AQP1*, *NROB1*, *FGF10*, *BMPR1B*, *IGF1*, *BMP2*, *AR*, *FSHR*, *CYP17A1*, *LHCGR*, *CYP11A1*, *HSD3B1*, *SMAD7*, *LOC416998* (corresponding to NALP1 protein) *EGR1*, *PGR* and *TIMP3*). Representative expression profiles of this cluster are sown in Figure 3 (see *BMP2* and *AR*). Similarly, seven genes (*NROB1*, *IGF1*, *FSHR*, *CYP11A1*, *HSD3B11*, *EGR1* and *PGR*) presented higher RE in later period compared to earlier period in the testis samples, and *NOG* expression increased during development but only in testis samples. In addition, several of the genes forming cluster 2 were preferentially expressed in the ovary compared to the testis. *FSHR* was significantly upregulated in the ovary in both periods. The RE of eight genes was significantly higher in the ovary compared to the testis in the later period (*FGF10*, *BMPR1B*, *BMP2*, *AR*, *CYP17A1*, *LHCGR*, *SMAD7*, and *LOC416998* (corresponding to NALP1 protein)).

Cluster C3 (Figure 2) contained 21 genes preferentially expressed in the ovary compared to the testis. Of these, *MHM*, *FOXL2*, *CYP19A1* and *HSD17B4* exhibited a stronger expression in the ovary in the earlier as well as in the later period. Moreover, eleven genes were preferentially expressed in ovarian samples compared to the testis in later period (*ESR2*, *ADAMTSL1*, *GATA6*, *EMB*, *FGF9*, *BMP4*, *BMP7*, *FST*, *BMP3*, *GDF9* and *SMAD2*). Eight genes (*GNRH1*, *ESR2*, ADAMTSL1, *GATA6*, *EMB*, *HSD17B4*, *GDF9*, *JAK1* and *TGFBR2*) exhibited upregulation during ovary development. Of these, *GNRH1*, *EMB*, *JAK1* and *TGFBR2* were also more highly expressed in earlier testis samples compared to earlier ovarian samples. Interestingly, among the genes with a preferential RE in the ovary, five genes were downregulated in the testis during the later period (*ADAMTSL1*, *BMP4*, *BMP7*, *HSD17B4* and *BMP3*). The RE of one gene (*MHM*) increased during testis development. The representative expression profiles of this cluster are shown in Figure 3 (See *BMP3* and *HSD17B4*).

Cluster C4 (Figure 2) included twenty-six genes. These genes presented significantly preferential RE in the earlier samples (between day 5.5 and 7.5) compared to the later samples. Of these, eight genes exhibited higher earlier RE in both sexes (*ACVR2B*, *FRZB*, *ESR1*, *GATA5*, *GREM1*, *PAX2*, *PITX*2 and *GATA2*) (see *GREM1* and *PAX2* in Figure 3). Six other genes presented preferential earlier RE only in testis samples (*GAL*, *SST*, *GJA1*, *HOXA7*, *WNT4*, and *GJB1*). Furthermore, the RE of *FOXO3* increased during ovarian development, whereas that of *KIT* increased during testis development. It was also observed that some genes in this cluster presented a sexual dimorphism in their expression patterns. Of these, *NGFB*, *ESR1*, *GREM1*, *GAL*, *GJA1*, *HOXA7*and *FOXO1A* had sexually dimorphic expression profiles in the later period with significantly higher RE levels in the ovary. The RE of *WNT4* was higher in the testis at the earlier stages whereas it was higher in the ovary at the later stages. Furthermore, *KIT*, *BMP5*, *FSTL1*, and *ACVR2B* were more highly expressed in the testis at the later stages.

**Figure 4:**
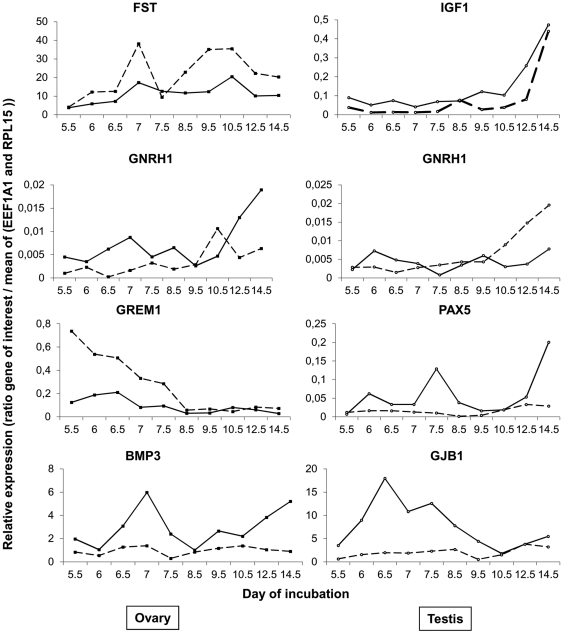
Expression profiles of some representative genes presenting asymmetric expression between left and right gonads. *FST* (Follistatin); *GNRH1* (gonadotropin-releasing hormone 1 (luteinizing-releasing hormone); *GREM1* (Gremlin 1, cysteine knot superfamily, homolog (Xenopus laevis)); *IGF1* (insulin-like growth factor 1 (somatomedin C)); *BMP3* (bone morphogenetic protein 3); *PAX5* (paired box 5); *GJB1* (gap junction protein, beta 1, 32 kDa). Male and female expression levels are represented by empty circles and black squares, respectively. Expression levels in the right and left gonads are represented by a dotted line and black line, respectively. Each dot represents the means of two different measurements corresponding to two different biological samples for the same male or female population or the same sampling date.

As described above we also compared gene expression in left and right ovary and testis samples. We could not determine a specific cluster of asymmetrically expressed genes. This was probably due to the fact that only 24 out of 110 genes displayed significantly asymmetric expression profiles in at least one left-right side comparison and that the patterns of these genes were not similar enough to be clustered together. *BARX1*, *SOX9*, *GDNF*, *NOG*, *CGNRHR*, *BMP3*, *ESR1*, *GAL* and *PITX2* were more highly expressed in the left ovary (See *BMP3* profile in Figure 4) than in the right and the expression of ten genes (*NR5A2*, *PAX5*, *DDX4*, *BMPR1B*, *IGF1*, *GNRH1*, *MHM*, *BMP3*, *PITX2* and *GJB1*) was greater in the left testis than in the right (See *GNRH1* profiles in Figure 4). At all stages, *PITX2* was preferentially expressed on the left of male and female gonads. Interestingly, *BMP3* was more highly expressed in the left ovary than in the right in the earlier and later periods. Moreover at the early ovary stages the expression of *CGNRH-R* was greater on the left, whereas the expression of *NR5A2* and *GREM1* was greater on the right (see *CGNRH-R* and *GREM1* profiles in Figure 4). At the later ovary stages, three genes were upregulated on the left (*SOX9*, *IGF1*, and *GAL*) whereas three other genes were more highly expressed on the right (*CYP11A1*, *FST*, *PAX2*) (See *FST* in Figure 4). At the earlier testis stages, five genes were upregulated on the left (*DDX4*, *IGF1*, *BMP3*, *PITX2* and *GJB1*) (see *GJB1* profile on Figure 4), and at the later testis stages *NR5A2*, *PAX5*, *IGF1*, *GNRH1* and *PITX2* were more highly expressed on the left.

### Whole-mount in situ hybridization

**Figure 5:**
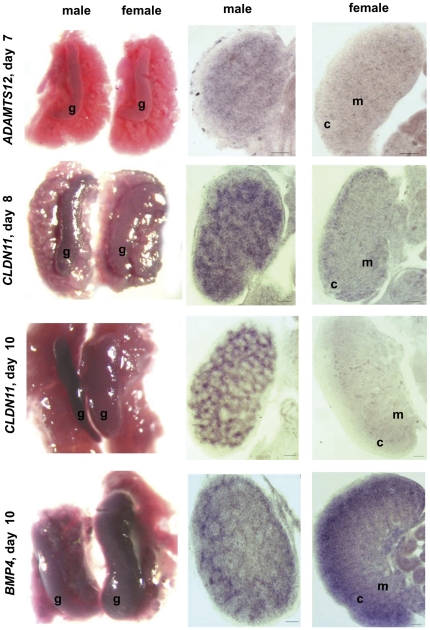
Expression of *CLDN11*, *ADAMTS12* and *BMP4* in left male and female embryonic gonads analyzed by whole mount in situ hybridization. Gonads with undelaying mesonephros and 50 µM gelatin sections of the same gonads are presented for each probe. g (gonad), m (medulla), c (cortex). Day of incubation is indicated in the figure for each analysis. Scale Bars = 50 µM.

We performed more detailed study of 3 genes (*CLDN11*, *ADAMTS12* and *BMP4*) by whole mount *in situ* hybridization (Figure 5). This analysis confirmed dimorphic expression of *CLDN11*, *ADAMTS12* and *BMP4* in male and female gonads, detected by real-time RT-PCR. *CLDN11* was highly preferentially expressed in the male gonads compared to the female gonads at day 8 and specifically expressed in the male gonads at day 10 (Figure 5). In the male gonads *CLDN11* expression was located in the testicular cords in the Sertoli cells. At day 8 faint *CLDN11* expression was observed in the cortex in the female gonads and it disappeared at day 10. In accordance with RT-PCR findings *in situ* hybridization analysis detected preferential expression of *ADAMTS12* in the testis at day 7, the time of histological differentiation of the gonads. The expression of *ADAMTS12* was located in the forming testicular cords. Based on RT-PCR findings the expression of *BMP4* in embryonic gonads becomes sexually dimorphic at day 10. *BMP4* was also more highly expressed in female than in male gonads at day 10 as detected by *in situ* hybridization. *BMP4* was expressed in the cortex and in the medulla but its expression was much greater in the cortex. In the male gonads *BMP4* was weakly expressed in testicular cords.

## Discussion

The chicken is a very appealing comparative model to study molecular mechanisms leading to testis or ovary formation. Indeed, among other model species in which the female is heterogametic, only the chicken is characterized by a high degree of differentiation of sex chromosomes Z and W, which may be considered as functional equivalents of the mammalian highly differentiated XY chromosome system where the specific small sex chromosome Y is shared by the male. Furthermore, gonad development in chicken embryos presents other features such as asymmetry of the female reproductive system and the sensitivity of gonad differentiation to estrogens. These particular features may have consequences on the regulation of genes that trigger sex determination and gonad differentiation in this species. Our results provide valuable information on the genes involved in chicken gonad differentiation. Using real-time RT-PCR we compared the expression pattern of 110 candidate genes and as already applied for the study of the genetic program of trout gonad development, these expression patterns were analyzed by a clustering method and displayed in a color coded matrix [13]. It was first of note, that the hierarchical clustering of the samples was consistent with the genetic sex and with the time of sampling and clearly discriminated two periods in male and female gonad differentiation. The distinction between early and late male samples revealed a delay compared to the onset of the histological sex differentiation which occurs between day 6.5 and 7 [14]. This can be explained by the fact that the fold change in the RE of several genes increases or decreases in male gonads by days 8.5–9.5. For instance, this is the case for *PAX2*, *GATA2*, *AMH*, *GREM1*, *PITX2*, *SST*, *SOX9*, *DMRT3*, *INHA*, *INHBA*, *INHB*, and *PDGFRA*. Secondly, hierarchical clustering of the genes discriminated four main gene clusters, three of which (C1, C2 and C3) contained genes exhibiting sexual dimorphism, and one cluster, C4, which mainly contained genes early regulated in both sexes, included new potential players (*GATA5*, *GATA2* and *PAX2*) of the development of the bipotential gonad. Similarly, some genes were upregulated in the later period in both sexes. This was the case for several genes from cluster 2 (e.g. *CYP11A1*, *HSD3B1*, *EGR1* and *PGR*) from cluster 1.3. These genes may be involved in gonad growth and steroid production.

Estrogens are known to have a critical role in avian sex determination and particularly in ovarian cortex development [12], [16]. Therefore, as expected, in cluster C3 with a specific ovarian signature, we found *CYP19A1*, an enzyme involved in estrogen production [11], [16], [17], [18] and its potential regulator *FOXL2* [19], [20].We also found in this experiment that the genes encoding other steroidogenic enzymes were preferentially expressed in the ovary, i.e. *CYP17A1* (C2) and *HSD17B4* (C3), consistent with the fact that the embryonic ovary produces more steroids than the testis [21], [22],[23]. One of the aims of this study was to find new potential regulators of enzymes involved in estrogen synthesis (in particular of CYP19A1) as well as estrogen targets. Of the genes upregulated in the developing ovary, *NGFB* (cluster C4) seems to be a good candidate because this neurotropin is involved in the reproductive function in mammals [24], [25], [26], [27], [28], [29], [30]. Indeed, *NGF* mRNA expression in rodents and humans begins before primordial follicle formation [25], [27], [28], and it is closely involved in ovary development [24], [26], [29], [30]. Furthermore, *NGF* seems to be a modulator of steroidogenesis in mammalian granulosa cells, decreasing progesterone production and increasing estradiol production by regulation of *FSHR* mRNA expression [31], [32], [33]. Thus *NGFB* could be involved in regulation of estrogen levels in chicken embryonic gonads. On the other hand, the upregulation of NGFB in the developing chicken ovary may be a consequence of *CYP19A1* upregulation since exposure to estradiol increases intraovarian *NGFB* expression in the postnatal rat ovary [34].

In the C1 cluster, we found genes that were specifically upregulated during testis differentiation. Some of them have been shown to be involved in vertebrate testis differentiation, including the chicken, (*SOX9*, *DMRT1* and *AMH*) [1], [8], [9], [13], [35], [36], [37], [38], and we used these genes to form the testicular cluster. Several genes joined the same cluster and were preferentially expressed in male gonads during the first stages of gonad differentiation (*TSKU*, *NOTCH1*, *F2R*, *FANCG*, *CFC1*, *DMRT3*, *GHR, ADAMTS12*, *INHA*, *CL11*, *PDGFRA*, *NR5A2*, *BMPR2 and LOC427192* (corresponding to *NIM1*)) suggesting that these genes would be good candidate early markers of male gonad differentiation. Six of these genes (*FANCG*, *CFC1*, *ADAMTS12*, *DMRT3*, *GHR and LOC427192*) are Z linked, and thus their preferential testicular expression may be the consequence of double chromosome dosage. Based on the fold change in their expression levels between the embryonic testis and ovary this could be the case for *FANCG*. However for 5 other genes the significant fold change in their expression levels between the embryonic testis and ovary indicates rather their specific regulation than the effect of the double Z chromosome dosage. Among these testicular genes, *CLDN11* exhibited a highly conserved developmental expression profile. As demonstrated by *in situ* hybridization in this study *CLDN11* was specifically expressed in Sertoli cells during testis differentiation in the chicken similar to mouse [39]. *CLDN11* is essential for tight junction formation and blood-testis barrier integrity. Absence of *CLDN11* results in male sterility [40]. *FANCG* (Fanconi anemia complex G) is interesting because it is a member of a complex formed by FANCA, FANCC and FANCG, known to regulate germ cell proliferation in the mouse [41]. Another gene, *ADAMTS12* (a desintegrin and metalloproteinase with thrombospondin type 1 motifs), is particularly interesting because, in addition to its role in the remodeling of the extracellular matrix and angiogenesis, this protein has been demonstrated to be an inhibitor of *SOX9* expression during in vitro chondrocyte differentiation [42]. Similarly *ADAMTS12* may be a modulator of *SOX9* expression in developing chicken gonads since in this study *ADAMTS12* expression was detected in forming testicular cods at the time of chicken sex differentiation. *LOC427192* encodes a Serine/Threonine Kinase named NIM-1, which is a member of the AMPK-related kinase family that also includes testis specific genes SNRK and TSSKs [43], [44], [45]. This gene is conserved in the chimpanzee, dog, cow, mouse, rat, chicken, zebrafish and *C.Elegans*, but little is known about its functions; NIM-1 expression has been detected in many rat tissues but significant levels of NIM1 activity were observed only in the brain and in the testis [44]. This gene may thus have a specific role in chicken testis differentiation and function.

One particularly interesting finding of this study was that several members of TGFβ family exhibited sexually dimorphic expression profiles. Indeed, several Bone Morphogenetic Proteins (BMPs) were more highly expressed during chicken ovary development and clustered with *CYP19A1*. This was the case for transcripts of several BMP signaling molecules (*BMP3*, *BMP4* and *BMP7*). Similarly, in cluster 2, that also contained genes whose RE was higher in the ovary (e.g. CYP17A1), we also found transcripts of BMP signaling molecule (BMP2), of one BMP receptor (BMPRIB) and of one inhibitory SMAD (SMAD7). It has already been shown that *BMP7* is early expressed (day 4.5/5) in the chicken gonadal mesemchyme, becoming female specific from day 8 in the left and right medullae [46], consistent with our results. Similarly, BMP2 is overexpressed in the mouse embryonic ovary and BMP7 in the trout embryonic ovary. Nonetheless, in contrast to findings in the chicken, *BMP7* is more highly expressed in the mouse testis from day 10.5 [47], and *BMP4* expression is not sexually dimorphic in trout embryonic gonads [13]. All these results obtained in different vertebrates thus show that the sexual dimorphism of *BMP* expression is not totally conserved. These BMPs may be involved in germ cell proliferation, as demonstrated in murine testis cell culture where BMP2 and BMP4 exhibited a mitogenic effect on germ cells [48], [49]. In addition to this potential role, BMPs may be involved in the regulation of steroid production, as shown in mammalian and hen granulosa cells [50], [51]. Moreover, GDF9 (another TGFβ family member) also belonging to this ovarian cluster, is an oocyte-specific factor in mammalian early-stage follicles and seems to be essential for further follicle progression [50]. Furthermore this factor also regulates steroidogenesis by inhibiting *FSHR* expression [52]. Similarly to our findings, *FST* has been found to be overexpressed in the mouse ovary at embryonic day E12.5, which is the time of gonad differentiation [53], [54]. Furthermore, in mammals, FST acts downstream from WNT4, in order to inhibit the coelomic vessel formation that is male-specific. In the chicken, in contrast to the mouse, there is no male-specific vessel, suggesting another role for FST in ovary development. However, in the mouse, FST is also involved in maintaining germ cells in the cortical region [55]. FST is known to neutralize BMP activity by non-competitive receptor binding [56], [57] and thus FST may regulate BMP activity in chicken ovary development.

In contrast to the preferential ovarian expression of several *BMPs* during gonad development found in this study, genes encoding other members of the TGFβ family including subunits α, βA and βB of inhibin were upregulated in the developing testis. These subunits are involved in the formation of inhibins (αβA or αβB) or activins (βAβA, βAβB or βBβB). This is the first report of early sexually dimorphic expression of the *βB and* α *inhibin* subunits during chicken testis differentiation. Sexually dimorphic expression of inhibin subunit α in chicken embryonic gonads has been reported previously at later stages [58]. In the chicken, inhibin secretion by the testis has been shown to be higher than that by the ovary [59], [60]. Inhibin A and activin A have been found to modulate androgen synthesis in testicular cells from 18- day-old chicken embryos *in vitro* [60]. In mammals, sexual dimorphism in inhibin production and secretion has been found in the rat [61], sheep [62], and bovine [63] as well as in humans and nonhuman primates [64]. In the mouse, activin B has been shown to contribute to the formation of the coelomic vessel, a male-specific artery critical for testis development [65]. However, as mentioned above, this particular vasculature structure is absent in the chicken embryonic testis. Moore et al. hypothesized that inhibins and activins could also regulate interstitial cell functions and increase spermatogonia proliferation [66]. *CFC1*, another Z-linked gene upregulated in the testis throughout the developmental period, encodes the co-receptor of another member of the TGFβ family (nodal) which is known to be largely involved in vertebrate embryonic patterning, including germ layers and left-right axis specification [67]. In addition to its role as nodal co-receptor, CFC1 has also been shown to antagonize activin signaling by binding to the activin-ACTRIIA/IIB complex and preventing its interaction with the ACTRIB receptor [68]; it might thus regulate activin activity in the developing testis.

This study also highlights many genes expressed asymmetrically between left and right developing gonads. The absence of a specific cluster for these genes could be explained by the fact that they are asymmetrically expressed at different periods of gonad development and in different sexes, and thus only a small number of genes exhibited similar asymmetric expression profiles. Moreover, the clustering is mainly constrained by the differences between gene expression levels between ovarian and testis samples and between early and later samples. As expected, we found that *PITX2* was more highly expressed in left gonads than in right gonads in both sexes. PITX2 has been shown to be involved in the asymmetric development of the ovary [69]. Furthermore, several other genes were specifically upregulated in the left ovary (*IGF1*, *CGNRH-R*, *BMP3*, *PITX2* and *GAL*) or in the left testis (*NR5A2*, *PAX5*, *DDX4*, *IGF1*, *GNRH1*, *BMP3 and PITX2*). The presence of genes differentially expressed between the right and the left male gonads, which both become functional testes, could reflect the primary asymmetric program present in the gonads of both sexes. Indeed, *PITX2*, which is essential for the establishing the asymmetry, is overexpressed on the left side in both sexes [69], [70], [71]. Moreover, the right testis retains the potential for regression, which could be induced by estrogens, while the left testis develops in an ovary or ovotestis under the same conditions [16], [18], [72]. The genes preferentially expressed in the left female gonad which will become a functional ovary, may have a role in the development of the cortex, which occurs only in the left side. Indeed, genes whose expression is restricted to the medulla, as for example those encoding steroidogenic enzymes or *FOXL2*, do not exhibit significant asymmetry in their expression patterns. However, this potential role in the development of the cortex of female left side upregulated genes should be confirmed by their cellular localization. Moreover, some genes are specifically increased in the right ovary which regresses from day 8; this is the case for *NR5A2*, *NROB1*, *CYP11A1*, *EMB*, *FST*, *GREM1 and PAX2*. These genes could be markers of ovary digenesis.

To conclude, this study reports a picture of gene expression during chicken gonad development from undifferentiated gonads up to latest embryonic testicular and ovarian stages before hatching and reveals strong similarities between chicken gonad sex differentiation and that of other vertebrate species, as well as several specific features of this process in the chicken. This study also identified new candidate genes which could be potential actors of female and male pathways whose roles should be further investigated. Our findings also suggest that gonad differentiation in the chicken involves a complex interplay between different members of the TGFβ protein family whose precise function remains to be elucidated.

## Materials and Methods

### Ethics statement

All experiments were carried out with respect for the principles of laboratory animal care. Approval of the ethics committee is not necessary because in France experiments involving the chicken embryo are not considered as animal experiments.

### Animals

Fertilized eggs from White Leghorn chickens were incubated at 37.8°C and 40% humidity. Eggs were then removed every half day between day 5.5 and day 7.5, every day between day 7.5 and 10.5 and every two days between 10.5 and 19.5. Chicken embryos were quickly decapitated immediately after removal from the eggs and before any dissection.

### Tissue collection

At each stage, the left and the right gonads were dissected separately, put individually in RNAlater® (Ambion) and stored at −20°C. A small piece of extragonadal tissue from each embryo was dissected, frozen immediately in liquid nitrogen and stored at −80°C prior to sex determination. After sexing of embryos the gonads were pooled (10–25 gonads per pool depending on the stage) according to sex, stage and side. Two pools of gonads were combined for each time point analysis. Samples were stored in RNAlater® at −20°C until RNA extraction.

### Genetic sexing of chicken embryos

Genomic DNA was extracted according to Estoup et al. [73]. Small pieces of tissue were incubated for 2 hours at 55°C in 0.2 ml of 10% Chelex100 resin (Bio-Rad) containing 200 µg/ml. proteinase K. After inactivation of proteinase K by heating at 99°C for 10 min, samples were centrifuged at 10,000 g for 1 min. PCR sexing was performed on 2 µl of genomic DNA as described by Clinton [74].

### Total RNA extraction and Reverse Transcription

Total RNA was extracted using TRI Reagent (Euromedex) according to the manufacturer's instructions. RNA quality and concentration were determined using an Agilent 2100 Bioanalyser and the RNA 6000 labchip kit (Agilent technologies) according to the manufacturer's instructions. For cDNA synthesis, 2 µg of total RNA were treated with 1 unit of RQ1 RNase-free DNase (Promega) according to the manufacturer's instructions and then denatured in the presence of a mix of oligodT (250 ng) and random hexamers (62.5 ng) for 5 min at 70°C. Reverse transcription (RT) was performed first at 25°C for 10 min and then at 42°C for 50 min using Moloney murine leukemia virus reverse transcriptase in the presence of dNTP (0.5 mM) and RNAsine (RNAse inhibitor, 20 U). (All products were from Promega).

### Primer design

Chicken candidate genes were chosen according to a literature survey on the basis of their involvement in the sex differentiation cascade in vertebrates or more generally in embryogenesis and reproduction (Table S2). The chicken sequences resulted from searches in international public databases. New chicken genes were verified to be true orthologs of mammalian genes using a reciprocal blast hit strategy [75]. The physical location of genes identified on chicken chromosomes was retrieved from both mapview [76] and from blat search [77]. The primers were purchased from Eurogentec or from Sigma Genosis.

### Real time PCR

Real-time RT-PCR (qPCR) was carried out on an AbiPrism 7000 (Applied Biosystems). Reactions were performed in 20 µl with 200 nM of each primer and 5 µl of a 30× dilution of the RT reaction and the qPCR Mastermix plus for SybrGreenI (Eurogentec). *EEF1A* and *RPL15* were used as reference genes to normalize expression levels of genes assayed. Two independent runs were performed for each reference gene. For each set of primers, the efficiency of the PCR reaction (linear equation: y = slope+intercept) was measured in triplicate on serial dilutions of the same cDNA sample (pool of reverse-transcribed RNA samples). Real-time PCR efficiency (E) was calculated using the following equation: 
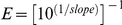
. Melting-curve analysis was also performed for each gene to check the specificity of RT-PCR products. The relative amount of the target RNA (R) was then determined using the following equation:
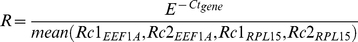
in which Ct is the cycle threshold and Rc1 and 2 are corrected relative reference gene expressions for runs 1 and 2 calculated as explained below. In order to take into account only fold changes in the expression levels of reference genes between samples but not the differences in the expression levels of reference genes (ref) in the sample, the expression levels of reference genes in each sample (sample_i_) were adjusted against the relative amount of EEF1A of run1 (EEF1A-run1) in one of two day 14.5 left female gonad samples (LFG-14.5) according to the equation:
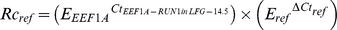
where 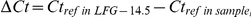


### Whole-mount in situ hybridization analysis

Whole-mount in situ hybridization was performed using digoxigenin-labeled riboprobes. For the probe synthesis the 434, 407 and 824 bp PCR fragments corresponding to *CLDN11*, *ADAMTS12* and *BMP4* respectively, were amplified from the embryonic gonad cDNA using the following pairs of primers: 5′-TCCTCTAGAAAACAAGCCCCA-3′, 5′-AGTGAGAAACCAACGGATTGC-3′ for *CLDN11* and 5′CACTGTTACGTATCGCAGCGT-3′ and 5′-AGGAGGCTACTTTCCGTGACA-3 for *ADAMTS12*, and 5′- CCCAAAGCCATGAACTCTTGC-3′ and 5′- CCACGATCCAATCGTTCCAA-3′ for BMP4. The PCR fragments were subcloned in the pCRII-TOPO vector (Invitrogen) and the plasmids were sequenced to confirm the presence of the correct insert. The riboprobes for *ClDN11*, *ADAMTS12* and *BMP4* were synthesized using an *in vitro* transcription kit (Roche Diagnostics) according to the manufacturer's instructions on the phenol-chloroform purified PCR products obtained by amplification of the inserts of interest from the plasmids using M13 reverse and M13 forward primers. The embryonic gonads at different developmental stages were dissected on ice with underlying mesonephros and fixed in 4% paraformaldehyde overnight. The hybridization procedure was performed as described previously [78]. For sectioning the gonads were embedded in phosphate buffered saline pH 7.5 containing 0.5% gelatin, 15% BSA, 2.2% glutaraldehyde, and 50 µM sections of the gonads were cut using MICROCUT H1200 (Biorade).

### Data Analyses

Real time PCR data were used to cluster genes and biological samples using a hierarchical clustering method [79] implemented in the T-mev4 software (MultiExperiment Viewer). The expression levels were first log-transformed and then the rows (genes) were median-centered and normalized, and the columns (samples) were normalized. A complete linkage method was applied on the similarity matrix based on the Pearson correlation coefficient in order to organize biological samples and to identify genes with a similar expression pattern. In order to distinguish between missing values due to PCR anomalies (gray colour on Fig. 3) and undetermined values due to a too weak gene expression level in the samples, we attributed a very low value (1×10^−13^) to these later samples before proceeding with the clustering. This was done only in the case of data obtained for CYP19A1 transcript measurement in male gonad samples, in which this gene is normally very weakly expressed, and therefore is often not determined by real time RT-PCR. As only the left ovary becomes completely functional, the comparisons of expression levels between the testis and the ovary, and between the earlier and later samples, were made using left gonads. We defined the comparisons of interest as: A) all left male and female samples; B) left “early” female and left “early” male samples; C) left “late” female and left “late” male samples; D) left “early” and left “late” female samples E) left “early” and left “late” male samples ; F) left and right female samples; G) left and right “early” female samples; H) left and right “late” female samples; I) left and right male samples; J) left and right “early” male samples; K) left and right “late” male samples. Differential analysis was performed on the dataset of the expression levels log-transformed using a linear model with one factor defined by the combination of the levels of the factors sex (male/female), side of the embryonic gonad (left/right) and developmental stages (early/late) with the Limma R package [80]. Contrasts of interest were tested and p values were adjusted for multiple testing using Benjamini and Hochberg's method to control the false discovery rate at the threshold fixed at 0.05 [15].

## Supporting Information

Figure S1 **Scatter plots of mRNA levels of 110 genes obtained from biological sample duplicates (Left ovary 1 vs Left ovary2; day 12.5).** Each black spot denotes a data point. The diagonal black line denotes X = Y identity
(TIF)

Table S1 **Relative expression values of 110 genes in embryonic chicken gonads detected by real time RT-PCR.** Each value in the table represents the expression level of the assayed gene detected by real time RT-PCR and normalized against the expression level of two reference genes *EEF1A* and *RPL15* as described in the section “Materials and Methods”. Each row corresponds to a gene; each column corresponds to a sample. The sex and the side of embryonic gonads in the samples are indicated. F = female, M = male, L = left, R = right. “#VALEUR!” indicates that the sequence was undetermined.
(XLS)

Table S2 **Names, symbols, accession numbers and chromosomal location of sequences of chicken genes and sequences of corresponding forward and reverse primers used in real time RT-PCR.**
(XLS)
