# Supplementary figures and images for: Gene Expression Profiling Reveals New Potential Players of Gonad Differentiation in the Chicken Embryo
Source: PLoS One. 2011 Sep 9;6(9):e23959. doi: 10.1371/journal.pone.0023959 (PMC3170287; doi:10.1371/journal.pone.0023959)

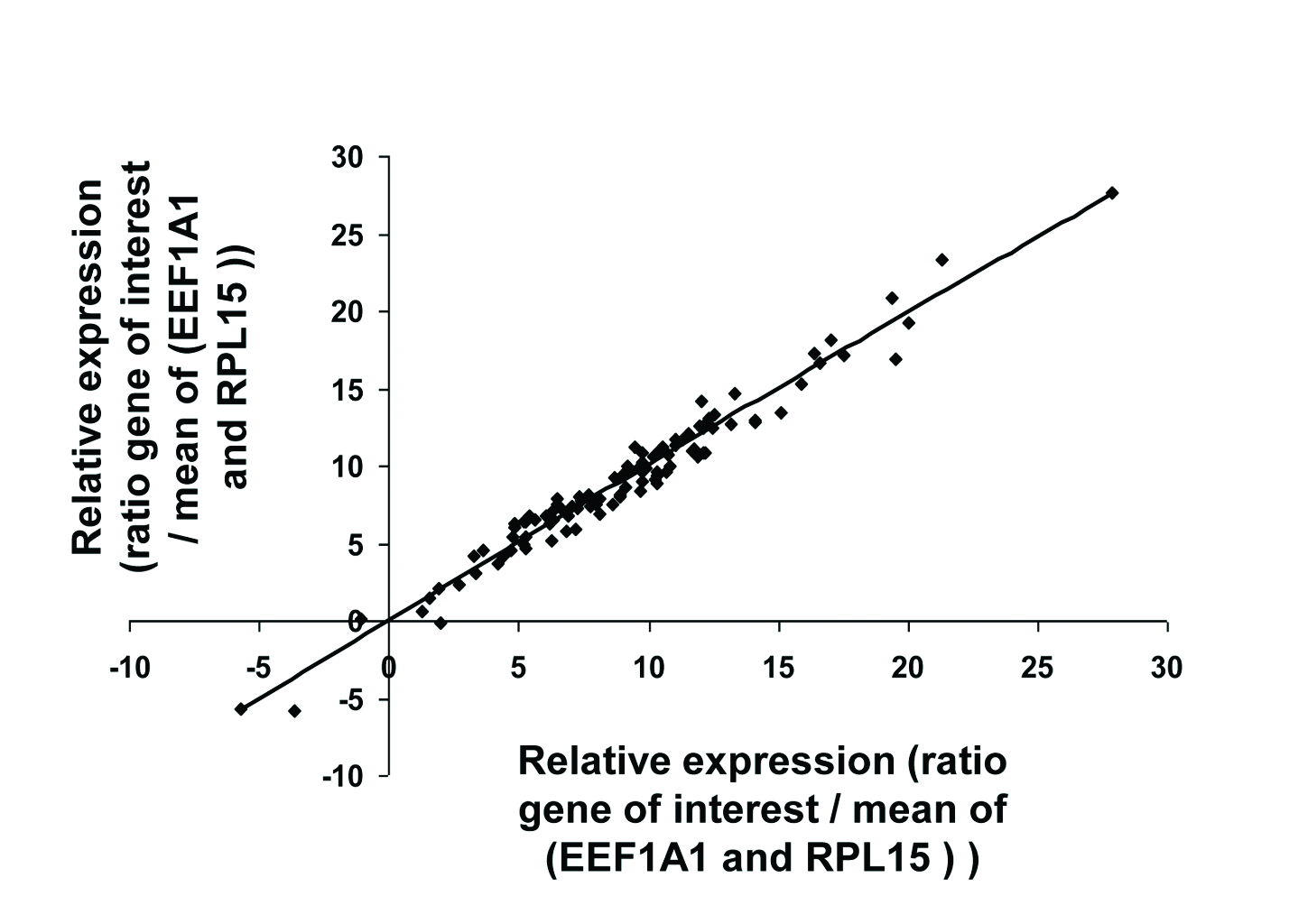

Supplement: Figure S1 — Scatter plots of mRNA levels of 110 genes obtained from biological sample duplicates (Left ovary 1 vs Left ovary2; day 12.5). Each black spot denotes a data point. The diagonal black line denotes X = Y identity (TIF) [file pone.0023959.s001.tif]
